# Machine learning predicts putative hematopoietic stem cells within large single-cell transcriptomics data sets

**DOI:** 10.1016/j.exphem.2019.08.009

**Published:** 2019-10

**Authors:** Fiona K. Hamey, Berthold Göttgens

**Affiliations:** Wellcome–MRC Cambridge Stem Cell Institute and Department of Haematology, University of Cambridge, Jeffrey Cheah Biomedical Centre, Cambridge Biomedical Campus, Cambridge, United Kingdom

## Abstract

Hematopoietic stem cells (HSCs) are an essential source and reservoir for normal hematopoiesis, and their function is compromised in many blood disorders. HSC research has benefitted from the recent development of single-cell molecular profiling technologies, where single-cell RNA sequencing (scRNA-seq) in particular has rapidly become an established method to profile HSCs and related hematopoietic populations. The classic definition of HSCs relies on transplantation assays, which have been used to validate HSC function for cell populations defined by flow cytometry. Flow cytometry information for single cells, however, is not available for many new high-throughput scRNA-seq methods, thus highlighting an urgent need for the establishment of alternative ways to pinpoint the likely HSCs within large scRNA-seq data sets. To address this, we tested a range of machine learning approaches and developed a tool, hscScore, to score single-cell transcriptomes from murine bone marrow based on their similarity to gene expression profiles of validated HSCs. We evaluated hscScore across scRNA-seq data from different laboratories, which allowed us to establish a robust method that functions across different technologies. To facilitate broad adoption of hscScore by the wider hematopoiesis community, we have made the trained model and example code freely available online. In summary, our method hscScore provides fast identification of mouse bone marrow HSCs from scRNA-seq measurements and represents a broadly useful tool for analysis of single-cell gene expression data.

## Highlights

• Single-cell gene expression profiling is widely used to profile hematopoietic cells.
• The best strategies for identifying stem cells currently rely on flow cytometry.
• Location of HSCs in single-cell data with no flow cytometry information is challenging.
• The hscScore method provides fast identification of HSCs from single-cell RNA-seq data.

It has been more than 60 years since experiments first proved the existence of bone marrow cells capable of producing the whole blood system. In the following decades, multipotent hematopoietic stem cells (HSCs) have been the subject of many studies aimed at revealing the mechanisms controlling their function [1]. Strategies to isolate blood cells were developed following the invention of techniques to sort cells based on their expression of specific proteins. By isolating and transplanting different fractions of bone marrow, sorting strategies could be refined to enrich for populations passing the gold-standard stem cell assay of repopulation upon secondary transplantation into irradiated mice (for review, see Mayle et al. [2]). Once HSCs could be isolated it became possible to measure molecular properties of these cells.

However, it is well known that many of the surface marker-defined hematopoietic stem and progenitor (HSPC) populations are very heterogeneous in terms of both function and their molecular profiles 3, 4, 5. The field of hematopoiesis has therefore been at the forefront of exploring single-cell technologies. In particular, many studies have used single-cell RNA sequencing (scRNA-seq) to profile gene expression across hematopoietic populations [3,6, 7, 8, 9, 10]. This has provided insights into processes such as differentiation, ageing, and disease (for review, see Watcham et al. [11]).

Initial scRNA-seq studies were limited in throughput by the cost and difficulty of profiling large numbers of cells. However, newer technologies such as droplet-based scRNA-seq methods 12, 13, 14 are enabling generation of increasingly large data sets, with multiple studies capturing tens of thousands of cells from the blood system [9,15, 16, 17]. This has many exciting implications for hematopoiesis research, yet these technologies bring their own challenges. Our best strategies for identifying HSCs rely on measurements of cell surface marker proteins [18,19]. However, many scRNA-seq data sets do not incorporate these measurements. Even in those studies using technologies such as index sorting [20,21] or CITE-seq [22] to link protein and gene expression, the identification of HSCs is still dependent on the choice of markers measured in the experiment. Therefore, identifying potentially rare populations of HSCs in single-cell data remains a challenge.

To address this, we decided to develop an approach that could be easily applied to scRNA-seq data with the aim of identifying transcriptional profiles belonging to HSCs. Using annotated data from a previous study of mouse HSPCs [19], we tested a range of machine learning methods to score single-cell transcriptomes based on their similarity to HSC gene expression, and identified a model performing well across data from a range of different laboratories and technologies. Along with this article we provide freely available code and the trained model so that researchers can easily apply this tool to their own single-cell data sets.

## Methods

### scRNA-seq data sets

#### Model training data

Models were trained on data from Wilson et al. [19]. In this study, 96 HSCs (Lin^−^c-Kit^+^Sca1^+^CD34^−^Flt3^−^CD48^−^CD150^+^) from mouse bone marrow were profiled using the Smart-Seq2 protocol [23]. Cells were filtered to the same 92 cells that passed stringent quality control (QC) measures in the original publication. Wilson et al. used a classification approach to assign scores to each transcriptome representing its similarity to a population highly enriched for functional HSCs (Figure E1A, online only, available at www.exphem.org). Data were visualized using principal component analysis (PCA) coordinates from the original publication. Count data, HSC-scores, QC information and PCA coordinates can be downloaded from Zenodo (https://zenodo.org/, DOI: 10.5281/zenodo.3303783).

### Index-sorted HSPC data

Data profiling 1,654 HSPCs were published in Nestorowa et al. [6]. These data were generated with the same Smart-Seq2 protocol as the training data. After QC, 798 Lin^−^c-Kit^+^Sca1^−^, 701 Lin^−^c-Kit^+^Sca1^+^, and 155 Lin^−^c-Kit^+^Sca1^+^CD34^−^Flk2^−^ cells were retained, and the count data for these cells can be downloaded from Zenodo (DOI: 10.5281/zenodo.3303783). QC information can be obtained from the data website (http://blood.stemcells.cam.ac.uk/single_cell_atlas.html). Data were visualized using the diffusion map coordinates and cell type information downloaded from the same data website.

#### Dormant and active HSC data

This data set was described in Cabezas-Wallscheid et al. [24]. scRNA-seq data were generated using the Fluidigm C1 microfluidics device to profile HSCs (Lin^−^c-Kit^+^Sca1^+^CD150^+^CD48^−^CD34^−^) and the subset of these cells that were long-term label-retaining, described as dormant HSCs (dHSCs). Gene expression counts for these data were downloaded from ArrayExpress (E-MTAB-4547). For QC, cells with  < 50,000 mapped reads,  < 1,000 detected genes, or  > 30% of reads mapping to External RNA Controls Consortium (ERCC) spike-ins were excluded, as in the original publication. For visualization, expression data were filtered to the highly variable genes (HVGs) from the original publication (Supplementary Table 2 in Cabezas-Wallscheid et al. [24]). Cells were normalized to have total counts equal to the median counts per cell, and normalized counts were log(*x* + 1) transformed with the *scanpy.preprocessing.log1p* function. A diffusion map was calculated on these log-transformed values using 30 neighbors and the “gauss” method in the *scanpy.tools.diffmap* function.

### Smart-Seq2 data of multipotent stem and progenitors

Data profiling LT-HSCs (Lin^−^c-Kit^+^Sca1^+^CD150^+^CD48^−^), ST-HSCs (Lin^−^c-Kit^+^Sca1^+^CD150^−^CD48^−^), and MPPs (Lin^−^c-Kit^+^Sca1^+^CD150^−^CD48^+^) were described in Mann et al. [25]. Expression counts were downloaded from NCBI GEO (GSE100426). This study profiled cells from young (8–12 weeks) and old (20–24 months) mice, and under stimulated (LPS treated) and unstimulated conditions. For testing of the hscScore method, only unstimulated cells were used. QC was performed by removing cells with fewer than 2,000 detected genes. For visualization, HVGs were identified using the *scanpy.preprocessing.filter_genes_dispersion* function with default settings, and data were normalised and log-transformed as described above. PCA was calculated on the log-transformed counts.

#### Droplet-based c-Kit^+^ cells

Transcriptomes for 22,993 Lin^−^c-Kit^+^Sca1^+^ and 21,809 Lin^−^c-Kit^+^ transcriptomes were generated using the 10x genomics [14] droplet-based sequencing method as described in Dahlin et al. [15]. Data can be downloaded from https://gottgens-lab.stemcells.cam.ac.uk/adultHSPC10X/ and NCBI GEO (GSE107727). Lin^−^c-Kit^+^ cells from W^41^/W^41^ mouse bone marrow were profiled similarly with data available from the same online resources. Data were visualized using the force-directed graph coordinates calculated for the original publication.

#### Droplet-based multipotent progenitors

Rodriguez-Fraticelli et al. [26] describe the generation of inDrops [12] scRNA-seq data from mouse bone marrow for each of the LT-HSC (Lin^−^c-Kit^+^Sca1^+^Flt3^−^CD150^+^CD48^−^), ST-HSC (Lin^−^c-Kit^+^Sca1^+^Flt3^−^CD150^−^CD48^−^), MPP2 (Lin^−^c-Kit^+^Sca1^+^Flt3^−^CD150^+^CD48^+^), MPP3 (Lin^−^c-Kit^+^Sca1^+^Flt3^−^CD150^−^CD48^+^), and MPP4 (Lin^−^c-Kit^+^Sca1^+^Flt3^+^CD48^+^) fractions. Processed count matrices were downloaded from NCBI GEO (GSE90742), and QC was performed by excluding any cells with fewer than 1,000 detected genes. For visualization, PCA was calculated as described above, and then UMAP [27] coordinates were calculated using the *scanpy.tools.umap* function with default parameters.

### Data pre-processing

Before input into the model, count data were processed by gene filtering and normalization. The gene filtering retained genes in one of three sets: (1) all protein-coding genes, (2) HVGs, or (3) MolO and NoMO gene sets. For option 1, only non-mitochondrial genes annotated as “protein_coding” in the Ensembl Version 81 annotation [28] were retained. For option 2, HVGs were calculated on normalised counts of all protein-coding genes (normalised using the *scanpy.preprocessing.normalize_total* function with default parameters). These normalised counts were log(*x* + 1)-transformed, and the HVGs identified with the *scanpy.preprocessing.highly_variable_genes* function with default parameters. Raw count data were filtered to this set of HVGs for input into the model. Option 3 retained the genes from Wilson et al.’s Supplementary Table 3 annotated as either MolO or NoMO genes [19]. These genes were those with significant correlation with the HSC-score assigned to each cell (adjusted *p* value  < 0.1, Benjamini–Hochberg correction for multiple testing).

After feature selection, count data were normalized on the selected genes using one of two alternatives: (1) rank normalization or (2) total count normalization. For rank normalization, expression in each cell was replaced by a vector representing the expression values ranked within that cell. Genes with identical counts were replaced with their average rank. For option 2, normalization was performed with the *scanpy.preprocessing.normalize_total* function to normalize each cell to have the same summed counts. This number of counts was set to be the median number of counts for the Wilson et al. data across the gene set of choice. Total count-normalized data were then log(*x* + 1)-transformed.

### Model training

To identify optimal parameters for each type of model, a search over parameters was performed using the *sklearn.GridSearchCV* function with fivefold cross-validation. Parameters explored for each model can be found in Supplementary Table E1 (online only, available at www.exphem.org). Before training, 25% of the data were held back as a test set, and the remaining 75% were scaled using the *sklearn.StandardScaler* function and then (optionally) PCA-transformed. The optimal parameters identified by the grid search are listed in Supplementary Table E2 (online only, available at www.exphem.org), along with the model *R*^2^ scores for each cross-validation fold, the mean and standard deviation of these scores, and the score of the trained model on the unseen test data. After the optimal parameters were obtained the models were retrained on the whole data set using these parameters.

### Plotting

Plotting was performed in python using either *scanpy* [29], *seaborn,* or *matplotlib* functions.

### Clustering and cell cycle scoring

Leiden clustering [30] was performed using the *scanpy.tl.leiden* function with resolution equal to either 1.0 for lower-resolution clustering or 1.5 for higher-resolution clustering. Before clustering, data from Nestorowa et al. were normalised using the *scanpy.preprocessing.normalize_total* function and log(*x* + 1)-transformed, and then HVGs were identified with the *scanpy.preprocessing.highly_variable_genes* function. PCA was calculated on the HVG values and the top eight principal components used for input to the clustering. Cell cycle scoring was performed by using the *scanpy.tl.score_genes_cell_cycle* function with S-phase and G2/M-phase genes downloaded from Macosko et al. [13].

### Code availability

Scripts for identifying model parameters and producing plots in this article are hosted on GitHub (https://github.com/fionahamey/hscScore). The trained model can be downloaded from Zenodo (DOI: 10.5281/zenodo.3332150). An example notebook on applying the model to new data is also hosted on GitHub.

### Software versions

Versions of all software used can be found in the Supplementary Material (online only, available at www.exphem.org).

## Results

### Linked stem cell function and gene expression data can be used to train models to identify HSCs

**Figure 1:**
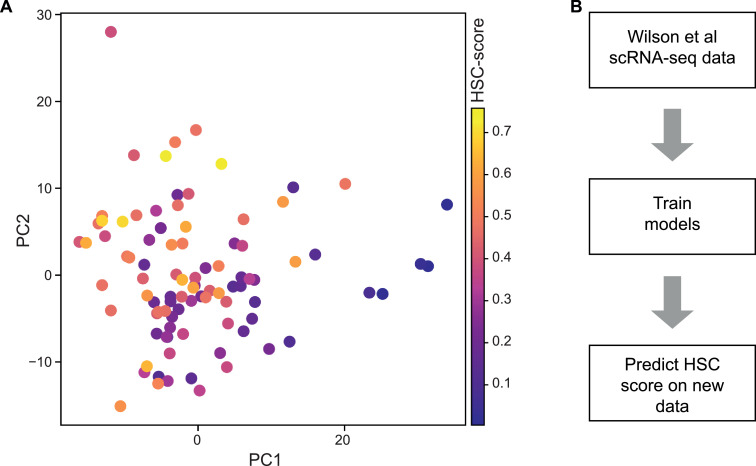
Predicting HSC identity in single-cell gene expression data sets. (A) Data from Wilson et al. [19] were used as training data for models predicting HSC identity in scRNA-seq data sets. In this study, 92 transcriptomes of HSCs were assigned a value, the HSC-score, where a higher HSC-score represents greater similarity to transcriptional profiles of functionally validated HSCs. (B) Outline of the training process for building the HSC prediction tool.

As our aim was to identify HSCs, we first required data where it was already known which transcriptomes belonged to these cells. This annotation could be done using surface marker expression, but even the purest HSC strategies still contain only up to 70% functional stem cells [19]. Therefore, we chose a data set of HSCs that were profiled as part of a study in which these cells were annotated with an HSC-score based on their gene expression [19]. This score represented each cell's transcriptional similarity to a highly homogeneous population of HSCs (Figure 1A; Supplementary Figure E1A). In this previous work, cells profiled using scRNA-seq were index-sorted to measure 11 flow cytometry parameters. To establish a link between the HSC-score and the functional output of a stem cell, single-cell transplantation assays were performed in which the same 11 flow cytometry parameters were recorded for each of the transplanted cells. On the basis of these shared parameters, dimensionality reduction was used to show that the repopulating HSCs in the single-cell transplantation experiments possessed surface marker profiles similar to those of the high-HSC-score cells. Therefore, this study established the correlation between having a high HSC-score and giving a positive readout in a transplantation assays designed to test for stem cell function [19]. Here, our aim was to use these scored transcriptomes to train models to predict the HSC-score of cells from new data sets (Figure 1B). To find the most suitable type of model for this prediction, we trained a number of different machine learning methods (linear regression, random forest regression, nearest-neighbor regression, support vector regression, and multilayer perceptron [MLP] regression) and scored the performance of each method on a test subset of the data (Supplementary Figure E1B). Model parameters were fitted using a grid search approach with fivefold cross-validation and then models were tested on unseen test subsets to assess their accuracy in predicting the HSC-score.

### Using a select subset of genes for training produces the most accurate models

Before training any models it was first necessary to define a pipeline for processing any scRNA-seq data set given as input to the model. In particular, it was important to choose analysis steps that would allow comparison of data across different experiments. Although scRNA-seq can measure thousands of genes per cell, the majority of genes detected across a data set have very noisy expression. To avoid obscuring biological variation in the data, often only a set of so-called highly variable genes (HVGs) that exceed a certain level of variance are used for analysis [31]. To explore the effect of gene set choice we decided to test models on three different gene sets: all protein-coding genes, HVGs, and the set of “MolO” and “NoMO” genes defined by Wilson et al. [19] (Supplementary Table E3, online only, available at www.exphem.org). Wilson et al. correlated the expression of all genes with the HSC-score within their scRNA-seq data, and denoted genes with significant positive correlation with the HSC-score as “MolO” genes and those with significant negative correlation as “NoMO” genes. Further details of these three different gene lists used for training can be found under Methods. As well as the choice of gene set, we also chose to test different data normalization methods, similar to work aimed at predicting cell cycle state based on gene expression [32].

**Figure 2:**
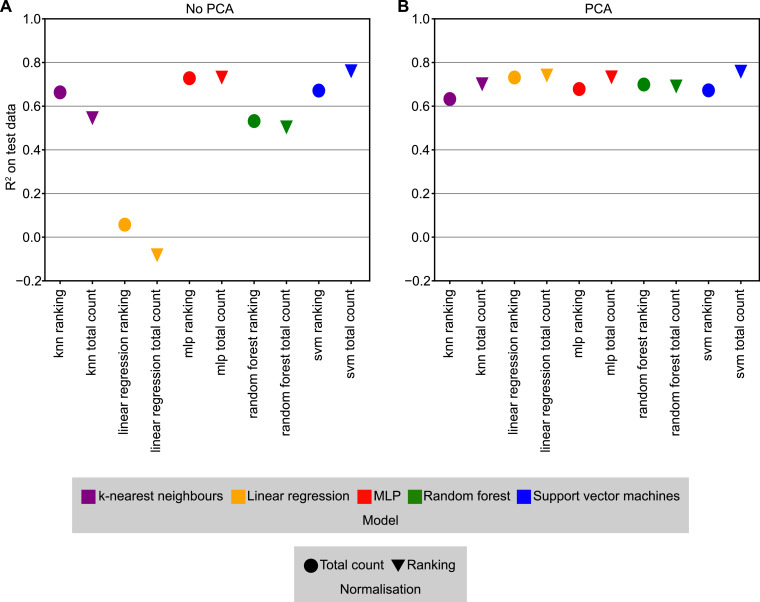
Trained models can predict HSC-score on unseen test data. *R*^2^ score of predicted compared with actual HSC-score on test subset of data for models trained with best identified parameters. Shape indicates normalization, and color, the type of method. Results are shown for models trained on raw counts (A) or PCA-transformed counts (B).

Many different normalization approaches have been applied to single-cell data, yet we needed one that would yield comparable results across multiple data sets. This requirement excluded many of the more sophisticated methods that share information across a sample to perform normalization [33,34]. We tested both total count normalization and a ranking normalization method (see Methods). Finally, we also tried training models on PCA-transformed data, reasoning that projecting new data into the PCA space of the training data could help to relate data sets from different technologies. Inspection of models trained across these combinations of pre-processing variables revealed that the best performing models were all trained using the MolO and NoMO genes (Supplementary Figure E2, online only, available at www.exphem.org). In general, models trained on the PCA-transformed data performed better on unseen data (Figure 2), although some models trained on untransformed counts were still amongst the highest scoring (Figure 2; Supplementary Figure E2).

### HSCs are successfully identified in a broad data set of blood stem cells and progenitors

**Figure 3:**
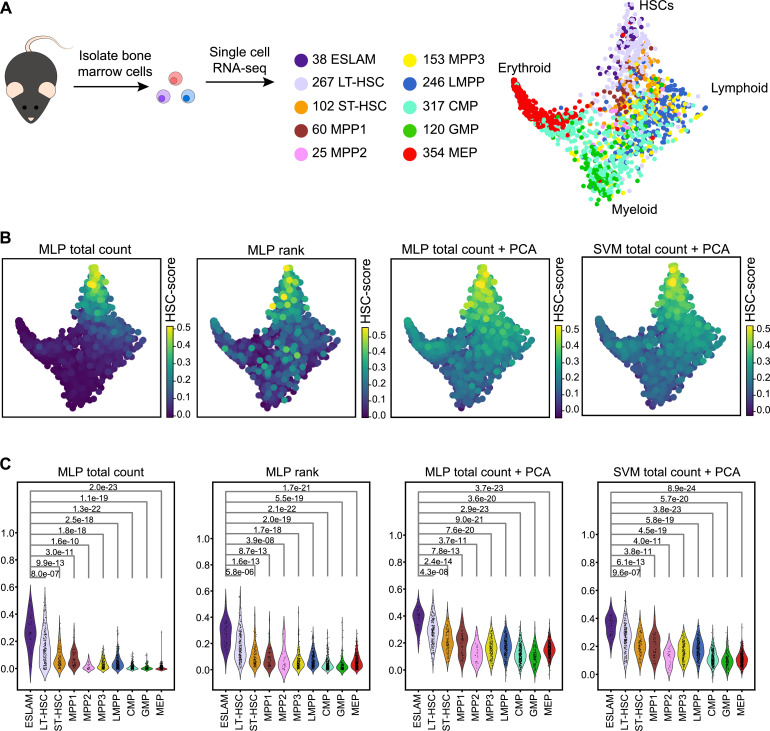
Top-performing models can identify HSCs in alternative data set profiling hematopoietic stem and progenitor cells. (A) Schematic of experiment from Nestorowa et al. [6] showing the number of cells for each surface marker-defined cell type in the scRNA-seq data set. Diffusion map dimensionality reduction is colored by surface marker cell type. (B) Diffusion map colored by the predicted HSC score from the top-performing models. Additional plots are shown in Supplementary Figure E3. Highest scores are seen in the region corresponding to phenotypic stem cells. (C) Violin plots showing distribution of scores across surface marker-defined phenotypes. *p* values indicate significance of pairwise tests between scores of each population in comparison to scores of ESLAM population, Wilcoxon rank-sum test. Additional plots are shown in Supplementary Figure E3. ESLAM=EPCR^+^ subset of HSCs; LT-HSC=long-term HSC; ST-HSC=short-term HSC; MPP=multipotent progenitor; LMPP=lymphoid-primed multipotent progenitor; CMP=common myeloid progenitor; GMP=granulocyte–macrophage progenitor; MEP=megakaryocyte–erythroid progenitor; MLP=multilayer perceptron; SVM=support vector machine.

After assessing the performance of the models on test data held back from the original data set, we next applied the highest scoring models to an alternative data set containing more than 1,600 HSPCs from mouse bone marrow using the same scRNA-seq protocol as the training data [6]. As this protocol was a plate-based method, cells were index sorted; hence single-cell transcriptomes could be retrospectively assigned to one of 10 different phenotypic cell types (Figure 3A). This data set contained 38 cells from the highly specific ESLAM (Lin^−^c-Kit^+^Sca1^+^EPCR^+^CD48^−^CD150^+^) HSC population [18] as well as more mature progenitor cells, allowing our models to be tested on a broader population than the training data. Diffusion map dimensionality reduction [35,36] revealed separation of HSCs from cells differentiating into erythroid, lymphoid, and myeloid lineages. For the majority of high-scoring models, high HSC-scores were localised to the top of the diffusion map in the region occupied by the ESLAM cells (Figure 3B; Supplementary Figure E3A, online only, available at www.exphem.org). HSC-scores were significantly higher in the ESLAM population when compared with other phenotypic cell types for a number of the models (Figure 3C; Supplementary Figure E3B, Wilcoxon rank-sum test, *p* values in figure). Overall, the MLP model with total count normalization and no PCA transformation gave the best distribution of HSC-scores across the data set, with high-scoring cells restricted largely to the ESLAM population. The score across all other populations was low, meaning this model was specifically highlighting the stem cells. As this combination of parameters mostly clearly highlighted the ESLAM cells that are enriched for functional HSCs in dimensionality reduction and violin plots, we therefore chose to carry this model forward for testing across a wider range of experiments and denote this prediction pipeline as hscScore.

One of the most widely used steps in the analysis of single-cell data is the application of clustering algorithms. Comparison of hscScore with a graph-based clustering approach [30] revealed that whilst clustering could identify a broad stem cell region, it nevertheless struggled to separate out the highest-HSC-score cells even with increased clustering resolution (Supplementary Figure E4A,B, online only available at: www.exphem.org). Clustering is also limited as it assigns cells into discrete groups, whereas hematopoietic differentiation may be better defined by a continuous representation [1]. Next, as there is known to be a link between cell cycle activity and repopulation capability of HSCs, we decided to compare scoring cells with hscScore to scoring cells by their expression of cell cycle genes [13]. In keeping with the reported quiescent nature of functional HSCs [37], we found a correlation between HSC-score and cell cycle score, with the group of cells most transcriptionally similar to HSCs having very low expression of cell cycle genes (Supplementary Figure E4C). This inverse relation between the HSC-score and cell cycle activity again supports the ability of hscScore to identify the stem cell population.

### hscScore locates HSCs in single-cell data sets produced by different technologies

**Figure 4:**
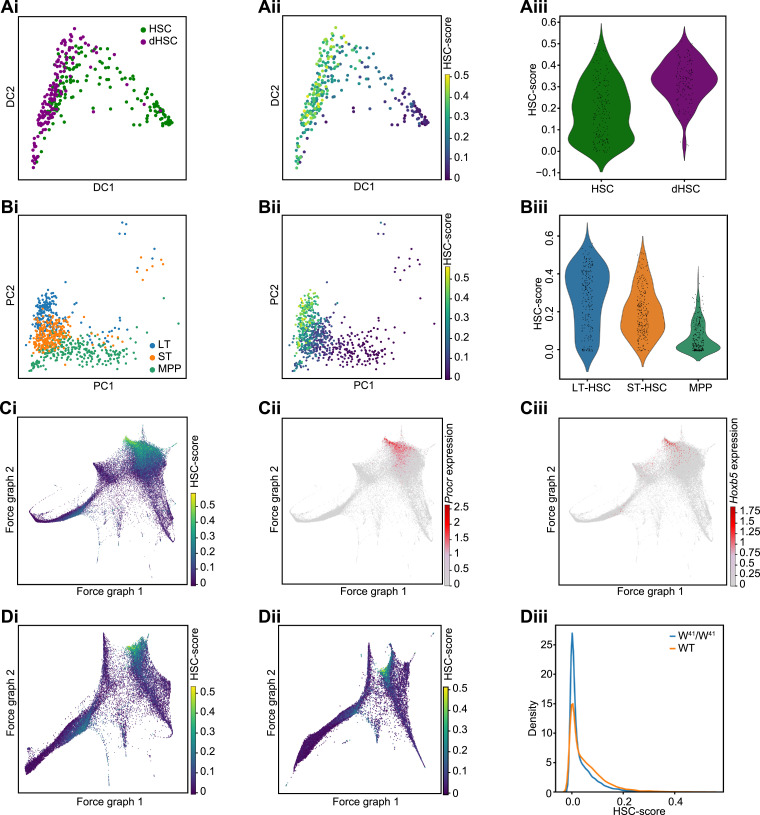
HSCs can be identified in data sets generated using different technologies. (A) Model performance on 316 HSCs from Cabezas-Wallscheid et al. [24]. Diffusion maps show data colored by cell sorting gate (i) and by predicted hscScore (ii). dHSC=dormant HSC. (iii) Violin plot shows HSC-score distribution over the dHSC and HSC gates. (B) Model applied to 718 SMART-Seq2 scRNA-seq profiles of stem and progenitor cells from Mann et al. [25]. PCA plots show the cell type (i) and predicted HSC-score (ii). (iii) The violin plot shows the score distribution across LT-HSC, ST-HSC, and MPP populations. (C) Application of top-performing model to droplet-based scRNA-seq data of 44,802 Lin^–^c-Kit^+^ bone marrow cells from Dahlin et al. [15]. Data are visualized using a force-directed graph colored by predicted HSC-score (i). Expression of HSC marker genes *Procr* and *Hoxb5* are shown in panels (ii) and (iii), respectively. (D) Force-directed graph of Lin^–^c-Kit^+^ bone marrow cells from wild-type (i) and W^41^/W^41^ (ii) mouse bone marrow colored by predicted HSC-score. (iii) Distribution of HSC-score across the wild-type and W^41^/W^41^ data sets.

To test the model's performance on data generated from a different laboratory and using an alternative scRNA-seq technology, we decided to investigate data from work by Cabezas-Wallscheid et al. [24]. In this study, the authors profiled dormant HSCs (dHSCs), a subset of HSCs that show long-term label retention in label-retaining assays. Previous work had shown that these dHSCs were enriched for repopulation potential and, therefore, represent a subset of HSCs containing a higher proportion of functional stem cells. 146 dHSCs and 170 HSCs were profiled using microfluidics scRNA-seq technology (Figure 4Ai). Diffusion map dimensionality reduction shows a progression from dHSCs to other cells within the HSC gate, which in the original study are shown to represent more “active” HSCs primed for cell cycle entry. Applying hscScore to these data revealed significantly higher (*p* =$\;1.1\;\times 10^{-19}$, Wilcoxon rank-sum test) scores in the dHSCs compared with the overall HSC population (Figure 4Aii, iii). We also tested our model on an additional data set containing long-term HSCs (LT-HSCs), short-term HSCs (ST-HSCs) and multipotent progenitor (MPP) populations [25] (Figure 4Bi). Again, highest scores were seen in the LT-HSC population, with lowest scores in the MPP populations (Figure 4Bii, iii; Supplementary Figure E4D).

Next, we wanted to see if our method would also work for higher-throughput single-cell gene expression methods such as droplet-based scRNA-seq. These approaches capture much larger numbers of cells but at least until now have had much lower sequencing depth. Additionally, many existing HSPC droplet-based scRNA-seq data sets do not have surface marker information for cells that would allow phenotypic populations to be identified. Application of hscScore to droplet-based data profiling of Lin^−^c-Kit^+^ mouse bone marrow cells [15] identified the highest-scoring cells in a specific region of the diffusion map (Figure 4Ci). Inspection of HSC marker genes *Procr* [38] and *Hoxb5* [39] revealed overlap between high HSC-score and expression of these genes (Figure 4Cii, iii). To examine another lower sequencing depth method, we calculated HSC-scores for LT-HSC, ST-HSC and MPP cells profiled using the alternative droplet-based method [26], and again the highest scores were found in the LT-HSCs (Supplementary Figure E5A, online only, available at: www.exphem.org).

We also asked how our method compared with a naïve approach of simply averaging MolO gene expression across cells, as we had previously found this to be useful in highlighting the HSC population [15]. Whilst we confirmed that this approach of averaging the expression of a specific gene set gave higher averages in the HSCs, these differences were not as clear as the hscScore model results. Instead, the average expression showed more of a gradient across HSPC populations (Supplementary Figure E5B–F), making it more challenging to clearly distinguish the HSCs with this approach.

### hscScore distribution is in keeping with lower proportion of stem cells in bone marrow of *Kit* mutant mouse

Finally, we applied our scoring method to previously published droplet-based scRNA-seq data from W^41^/W^41^ mouse bone marrow [15]. The W^41^/W^41^ mutation leads to reduced c-Kit signaling activity, and these mice have a lower proportion of stem cells [40,41]. We wanted to see if our approach could both detect stem cells in the mutant background and identify their shift in numbers. Dimensionality reduction on both wild-type and W^41^/W^41^ Lin^−^c-Kit^+^ cells showed very similar appearances and localization of the cells with high HSC-scores, verifying that this tool can be applied to these data from perturbed hematopoiesis (Figure 4Di, ii). The distribution of the HSC-score across the whole data set revealed the W^41^/W^41^ population had overall lower scores, in keeping with the reduction of HSCs within this mutant model (Figure 4Diii). The wild-type Lin^−^c-Kit^+^ population is expected to contain around 1% HSCs so we calculated the 99th percentile of the wild-type Lin^−^c-Kit^+^ hscScore. Only 0.56% of W^41^/W^41^ HSCs had a predicted score above this same threshold. This was in spite of the numerical range of scores being similar across these data sets ($-7.8\;\times 10^{-3}$ – 0.51 for W^41^/W^41^ and $-8.3\;\times 10^{-3}$ – 0.53 for wild-type cells). This shows that the hscScore method gives results in keeping with the reduced frequency of stem cells in the W^41^/W^41^ mouse model.

## Discussion

A rapidly growing number of studies use single-cell gene expression profiling to investigate the molecular state of blood stem and progenitor cells. One of the challenges when working with this type of data is to reliably identify the transcriptomes belonging to rare cell types. This is particularly relevant for those cell types conventionally defined by expression of specific cell surface marker proteins as many scRNA-seq data sets do not contain information on protein expression. In this work we trained and tested a range of predictive machine learning models to develop a tool to score single-cell gene expression profiles for their transcriptional similarity to a functionally pure population of HSCs.

It is well established that integrating or comparing scRNA-seq data from different sources can be difficult because of so-called batch effects arising from factors such as different experimental techniques [42,43]. We therefore tested our method across a number of data sets and identified a pipeline that performed well across scRNA-seq platforms with different sequencing depths. Optimal model performance was found when training on a small set of genes highly correlated with the HSC-score. We chose to include genes with both positive and negative associations to provide as much information as possible to distinguish between “good” and “bad” stem cells. The inclusion of these negatively correlated genes, as well as the fact that the hscScore model can learn specific weights for each gene, offers benefits over simply averaging the expression of a gene set. The flexibility in the MLP framework also allows varying weights across genes, meaning that there are different combinations of gene expression enabling a cell to get a high HSC-score.

We made efforts to ensure that our approach can be easily applied by other researchers, providing both the trained model and example code online. We envisage the hscScore method to be an easy step in the analysis of murine bone marrow scRNA-seq samples, enabling fast and reliable identification of HSCs in a data set. When the expected frequency of stem cells in a sample is known, it could be used to select a threshold for classifying cells based on their HSC-score, although this information will not be available for all data sets. In these cases, hscScore can still be used to reveal the most likely stem cells instead of being used for strict classification. Our hscScore approach also has the potential to be used as part of a pipeline for refining stem cell sorting strategies by identifying any genes that encode for surface marker proteins and have expression levels correlated with the HSC-score. With high-quality cell state annotation this approach could be applied to other systems. In particular, this would be worth exploring in systems where there are linked functional data and expression data, for example, through the expression of shared surface marker profiles. Of special interest to hematopoiesis, it would be interesting to try and extend this approach to identifying human HSCs, as a number of markers differ between human and mouse HSCs.

An exciting potential application of the hscScore method will be to compare data across different conditions, including genetic perturbations such as the W^41^/W^41^ mouse model explored here. A number of blood disorders affect stem cell behavior, and in such situations surface marker expression is commonly disrupted, making it unreliable to identify HSCs using conventional strategies. In particular, there are several mouse models in which an increase in the number of phenotypic HSCs but a decrease in the number of functional HSCs has been described. Where this decreased functionality is linked to transcriptional changes, a lower frequency of stem cells should be seen with hscScore. Being able to robustly identify HSCs within scRNA-seq data could therefore provide important new insights into disrupted hematopoiesis in these situations.

In summary, the hscScore model provides a fast and simple approach to identification of HSCs within scRNA-seq data sets from mouse bone marrow. This should provide a broadly useful tool for analysis of single-cell gene expression data, which we hope will be adopted widely by the community.

## Appendix Supplementary materials

Image, application 1

Image, application 2

Image, application 3
