# Supplementary material for: Machine learning predicts putative hematopoietic stem cells within large single-cell transcriptomics data sets
Source: Exp Hematol. 2019 Oct;78:11–20. doi: 10.1016/j.exphem.2019.08.009 (PMC6900257; doi:10.1016/j.exphem.2019.08.009)
Supplement: Supplementary file 1 [file mmc1.pdf]

Supplementary material for *Machine learning predicts putative haematopoietic stem cells within large single-cell transcriptomics datasets*

Fiona K. Hamey[a], Berthold Göttgens[a]

[a] *Wellcome - MRC Cambridge Stem Cell Institute and Department of Haematology, University of Cambridge, Jeffrey Cheah Biomedical Centre, Cambridge Biomedical Campus, Cambridge, CB2 0AW, UK*

Software information

The python modules and their versions used for the analysis in this manuscript are given below:

- python== 3.7.3
- anndata==0.6.21
- leidenalg==0.7.0
- matplotlib==3.0.3
- numpy==1.16.4
- pandas==0.24.2
- seaborn==0.9.0
- scanpy==1.4.3
- scikit-learn==0.21.2
- scipy==1.2.1
- statsmodels==0.9.0
- umap==0.3.9

Supplementary tables

Table E1: Model training parameters. Input parameter ranges for GridSearchCV, which uses cross-validation to score each combination of parameters from the ranges specified and returns the best scoring model.

Table E2: GridSearchCV results. Results of running GridSearchCV. "Gene set" column indicates the set of genes used for training the model. All, all protein-coding genes; Variable, highly variable genes; Molo, MolO + NoMO genes. "Normalisation" column describes whether counts were normalised with ranking or total count method. "PCA" describes if the model was trained using PCA-transformed data. "Best_parameters" column has the highest-scoring combination of parameters returned by the parameter search. "cv_x_score" columns show the R^2 score for each cross-validation fold with the best parameters. "Average_score" and "SD" columns are the mean and standard deviation of these scores, respectively. "Score_on_test_data" column contains the result of applying the model with the best parameters to test data not used for training. *Table is included as separate file.*

Table E3: MolO and NoMO genes. Genes from Wilson et al. [1] annotated as MolO or NoMO genes. *Table included as separate file.*

Model	sklearn function	Parameter	Values
Random forest	sklearn.ensemble.Random-ForestRegressor	max_depth	[2, 5, 10, 15, None]
		min_samples_leaf	[1, 2, 5, 10]
		min_samples_split	[2, 5, 10]
		max_features	[5, 'auto', 'sqrt', 'log2']
Linear regression	sklearn.linear_model	fit_intercept	['True', 'False']
Support vector regression	sklearn.svm.SVR	kernel	['linear', 'poly', 'rbf', 'sigmoid']
		C	[0.1, 1.0, 10]
		gamma	[0.01, 0.1, 1.0, 10]
		degree	[2, 3, 4, 5]
		coef0	[0, -1, 1]
		epsilon	[0.1, 0.01, 1]
Nearest neighbors regression	KNeighborsRegressor	p	[1, 2]
		n_neighbors	[3, 5, 10, 15]
Multi-layer perceptron	MLPRegressor	activation	['identity', 'logistic', 'tanh', 'relu']
		hidden_layer_sizes	[(100, 50, 100), (100, 100), (100, 100, 100), (50, 50), (50, 50, 50), (50, 25, 50)]
		alpha	[0.1, 0.01, 0.001, 0.0001]
		solver	['lbfgs', 'adam']
PCA	sklearn.decomposition. PCA	n_components	[5, 20, 50]

Table E1: Model training parameters. Input parameter ranges for GridSearchCV, which uses cross-validation to score each combination of parameters from the ranges specified and returns the best scoring model.

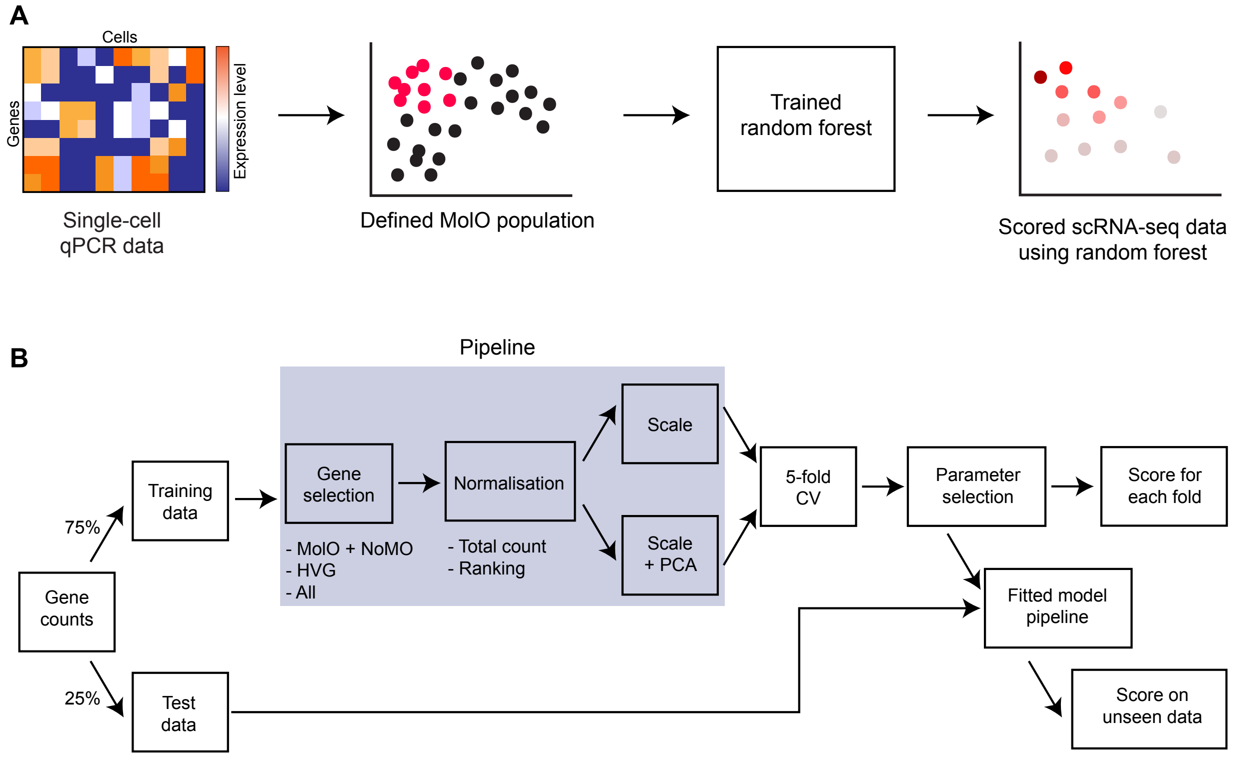

Figure E1: Overview of model training. (A) Schematic of how Wilson et al. [1] used single-cell qPCR data to define a population highly enriched for HSCs (the molecular overlapping (MolO) population). A random forest classifier to identify the MolO cells was trained on the expression profiles of these cells. scRNA-seq profiles of HSCs were then scored using this classifier, giving each one an HSC-score. These scored single-cell transcriptomes are then used as the training data for our hscScore method. (B) Summary of the process for training and scoring different types of models. The scored gene expression counts from (A) were used to train different types of predictive models. The workflow gives a trained model pipeline which can then be applied to new datasets.

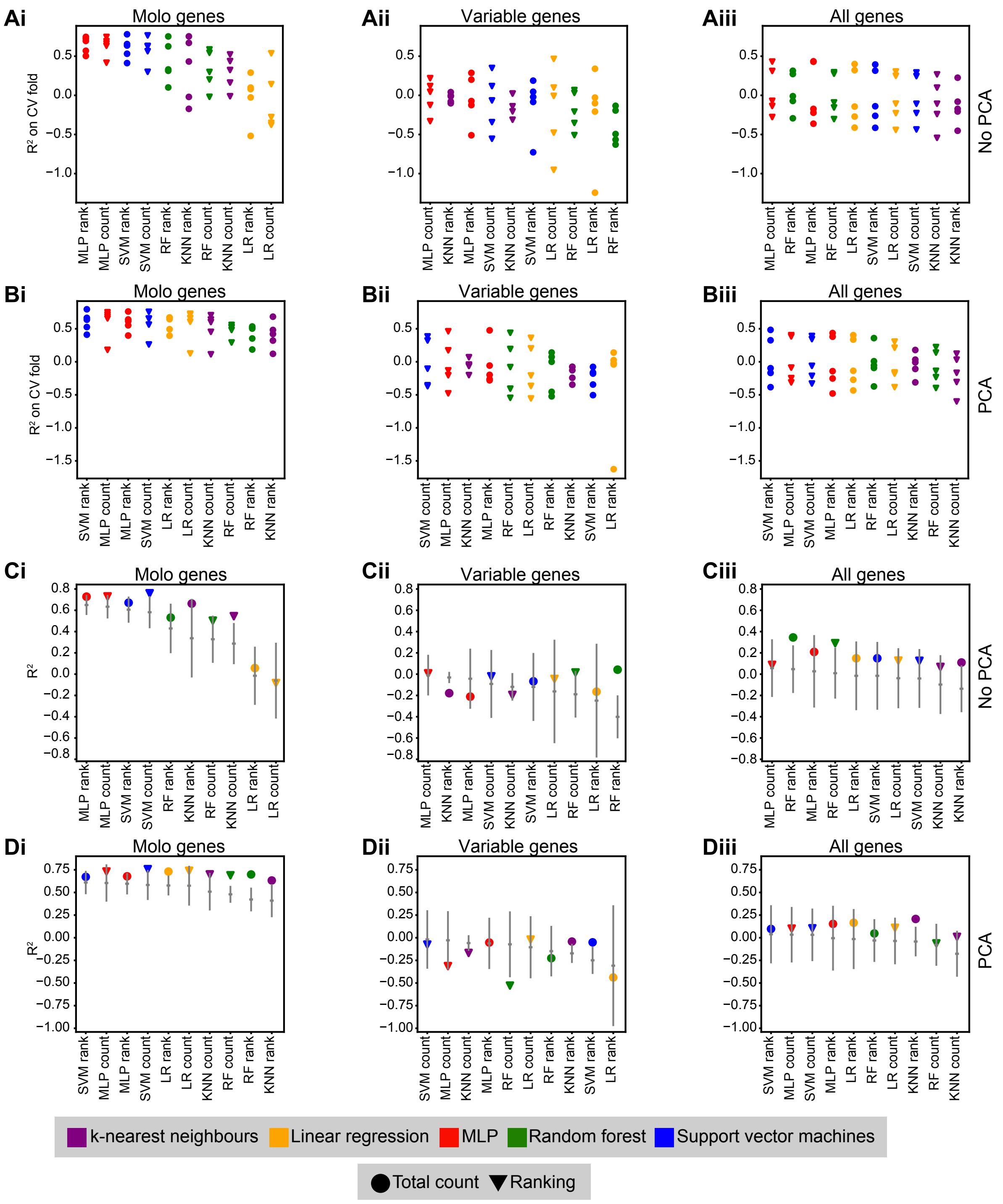

Figure E2: Performance of different predictive methods across alternative gene sets and normalisations. (A, B) R^2 of each fold for 5-fold cross validation (CV) on training data. The colour of each point corresponds to the type of model and the shape corresponds to the normalisation used (total count or ranking). (A) shows the results for models fitted with no PCA-transformation and (B) for models trained on the top 50 principal components. (C, D) R^2 of trained models applied to test data. Error bars show mean and standard deviation of CV results from panels above. (C) shows the results for models trained without PCA-transformation and (D) for models trained on the top 50 principal components. Results are shown for training on (i) MolO + NoMO genes (ii) highly variable genes and (iii) all protein-coding genes.

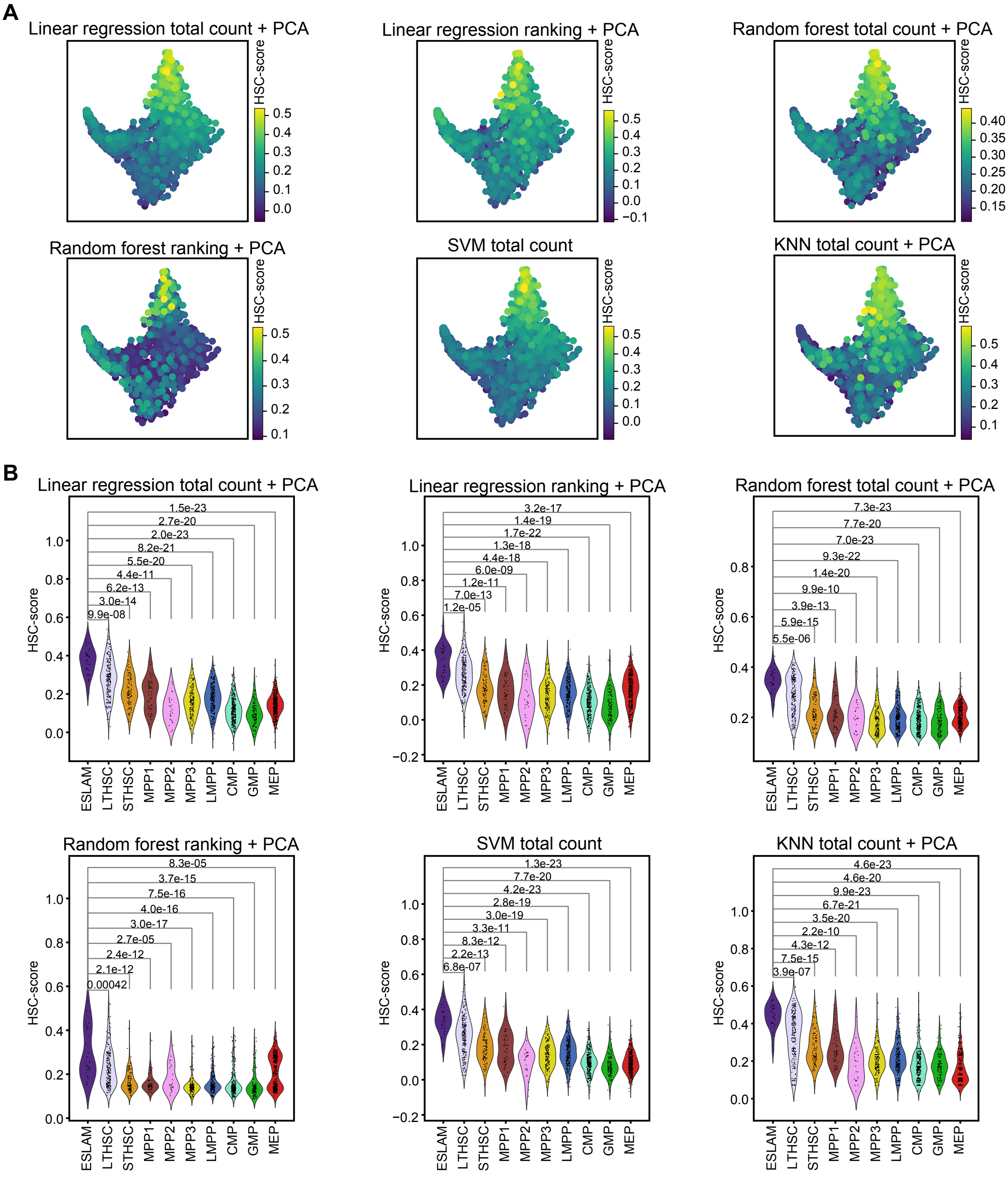

Figure E3: Model performance on data from Nestorowa et al. [2]. (A) Diffusion map plots of 1,654 HSPCs from mouse bone marrow coloured by predicted HSC-scores using models trained on the Wilson et al. data [1]. (B) Violin plots of HSC-scores across cell surface marker-defined cell types of the Nestorowa et al. data. P-values indicate significance of pairwise tests between scores of specific populations in comparison to scores of the ESLAM population, Wilcoxon rank-sum test. SVM, support vector machine; KNN, k-nearest neighbours.

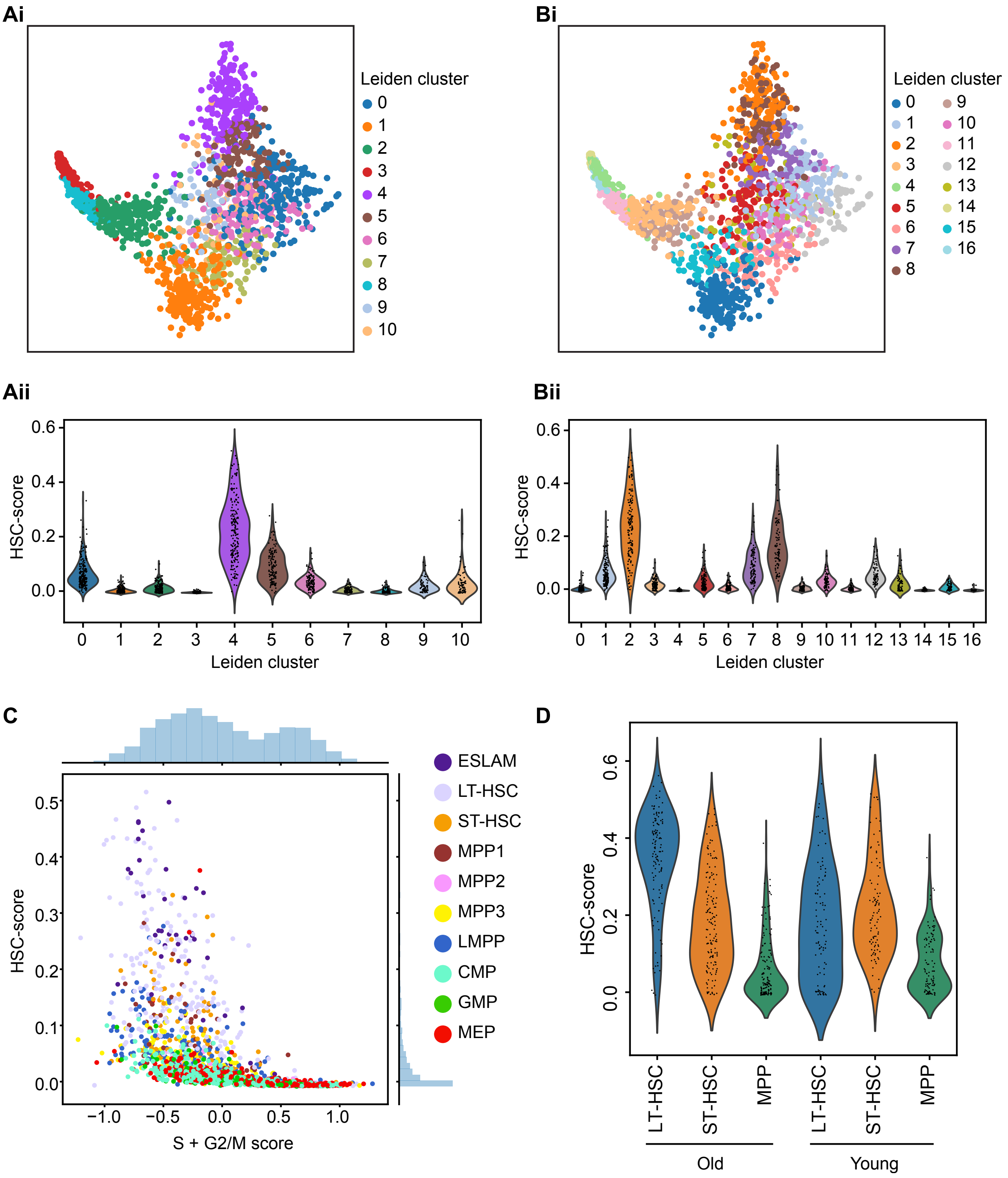

Figure E4: Comparison of hscScore to clustering and cell cycle scoring. (Ai) Diffusion map of Nestorowa et al. data [2] coloured by Leiden clustering with resolution=1.0. (Aii) Violin plot of predicted HSC-score across the Leiden clusters with resolution=1.0. (B) As in panel (A), but with resolution=1.5 for Leiden clustering. (C) HSC-score against sum of the S-phase and G2/M gene scores calculated on the Nestorowa et al. data. Cells are coloured by phenotypic cell type. Histograms to top and right of plot show the distribution of cells in the HSC-score vs cell cycle score space. Note that the majority of cells have very low HSC-score and so overlap in the plot. (D) HSC-score of unstimulated cells from Mann et al. [3] split by phenotypic cell type and age. Note that cells were placed in culture for 2 hours before single-cell sorting, and that young and old HSCs may respond differently to culture conditions making differences in the HSC-score between ages hard to interpret.

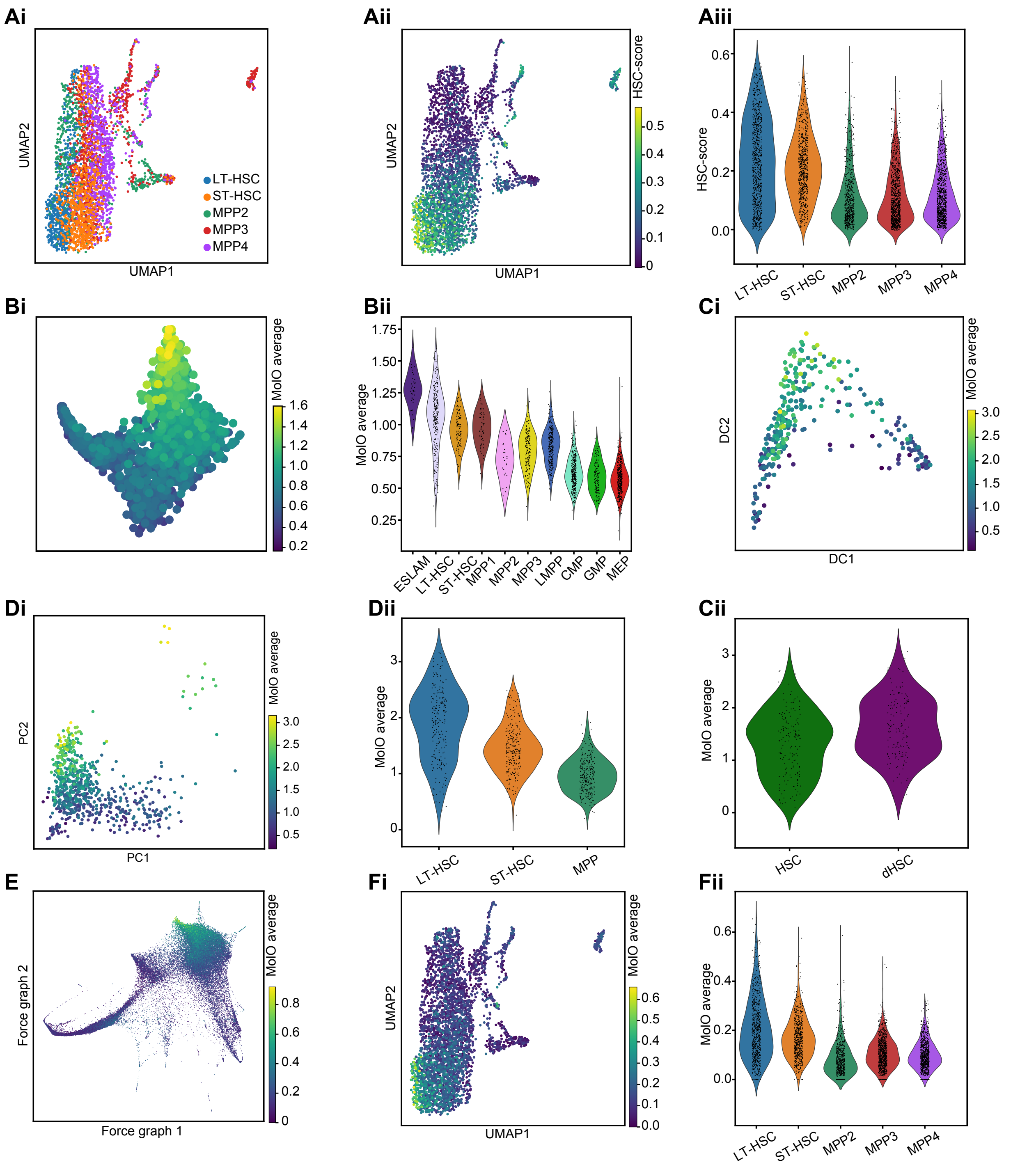

Figure E5: Comparison of hscScore to using average MolO gene expression to highlight HSCs. (A) Prediction of HSC-scores from Rodriguez-Fratecelli et al. [4]. (i) UMAP dimensionality reduction coloured by phenotypic cell type. (ii) UMAP coloured by predicted HSC-scores. (iii) Violin plot showing distribution of HSC-scores across phenotypic cell types. (B-F) Using average expression of MolO genes to highlight the HSC population. (Bi) MolO gene average plotted on diffusion map from Nestorowa et al. [2]. (Bii) Violin plot of MolO average across Nestorowa et al. phenotypic cell types. (Ci) MolO average plotted on diffusion map from Cabezas-Wallscheid et al. [5]. (Cii) Violin plot of MolO average across Cabezas-Wallscheid et al. dormant and overall HSCs. (Di) MolO average plotted on PCA from Mann et al. [3]. (Dii) Violin plot of MolO average across Mann et al. phenotypic cell types. (E) MolO average plotted on force-directed graph from Dahlin et al. [6]. (Fi) MolO average plotted on UMAP map from Rodriguez-Fraticelli et al. (Fii) Violin plot of MolO average across Rodriguez-Fraticelli et al. phenotypic cell types.

References

[1] N. K. Wilson, D. G. Kent, F. Buettner, M. Shehata, I. C. Macaulay, F. J. Calero-Nieto, M. Sánchez Castillo, C. A. Oedekoven, E. Diamanti, R. Schulte, C. P. Ponting, T. Voet, C. Caldas, J. Stingl, A. R. Green, F. J. Theis, B. Göttgens, Combined Single-Cell Functional and Gene Expression Analysis Resolves Heterogeneity within Stem Cell Populations, Cell Stem Cell (2015) 712–724doi:10.1016/j.stem.2015.04.004.

[2] S. Nestorowa, F. K. Hamey, B. Pijuan Sala, E. Diamanti, M. Shepherd, E. Laurenti, N. K. Wilson, D. G. Kent, B. Göttgens, A single-cell resolution map of mouse hematopoietic stem and progenitor cell differentiation, Blood 128 (8) (2016) e20–e31. doi:10.1182/blood-2016-05-716480.

[3] M. Mann, A. Mehta, C. G. de Boer, M. S. Kowalczyk, K. Lee, P. Haldeman, N. Rogel, A. R. Knecht, D. Farouq, A. Regev, D. Baltimore, Heterogeneous Responses of Hematopoietic Stem Cells to Inflammatory Stimuli Are Altered with Age, Cell Reports 25 (11) (2018) 2992–3005.e5. doi:10.1016/j.celrep.2018.11.056.

[4] A. E. Rodriguez-Fraticelli, S. L. Wolock, C. S. Weinreb, R. Panero, S. H. Patel, M. Jankovic, J. Sun, R. A. Calogero, A. M. Klein, F. D. Camargo, Clonal analysis of lineage fate in native haematopoiesis., Nature 553 (7687) (2018) 212–216. doi:10.1038/nature25168.

[5] N. Cabezas-Wallscheid, F. Buettner, P. Sommerkamp, D. Klimmeck, L. Ladel, F. B. Thalheimer, D. Pastor-Flores, L. P. Roma, S. Renders, P. Zeisberger, A. Przybylla, K. Schönberger, R. Scognamiglio, S. Altamura, C. M. Florian, M. Fawaz, D. Vonficht, M. Tesio, P. Collier, D. Pavlinic, H. Geiger, T. Schroeder, V. Benes, T. P. Dick, M. A. Rieger, O. Stegle, A. Trumpp, Vitamin A-Retinoic Acid Signaling Regulates Hematopoietic Stem Cell Dormancy, Cell 169 (5) (2017) 807–823.e19. doi:10.1016/j.cell.2017.04.018.

[6] J. S. Dahlin, F. K. Hamey, B. Pijuan-Sala, M. Shepherd, W. W. Y. Lau, S. Nestorowa, C. Weinreb, S. Wolock, R. Hannah, E. Diamanti, D. G. Kent, B. Göttgens, N. K. Wilson, A single-cell hematopoietic landscape resolves 8 lineage trajectories and defects in kit mutant mice., Blood 131 (21) (2018) e1–e11. doi:10.1182/blood-2017-12-821413.
